# Comparison of joint laxity measured using 60- and 80-N distraction forces and manual stress during robot-assisted total knee arthroplasty

**DOI:** 10.1186/s42836-026-00449-9

**Published:** 2026-11-10

**Authors:** Hiroshi Inui, Haruhiko Nakamura, Ryota Takei, Masaki Uchida, Kei Satou, Tetsu Yamashita, Risa Matsumoto

**Affiliations:** Department of Orthopaedic Surgery, Saitama Medical University, Kawagoe City, Saitama, 350-8500 Japan

**Keywords:** Total knee arthroplasty, Robotic-assisted surgery, Joint laxity, Soft-tissue balance, Interobserver reliability

## Abstract

**Background:**

Appropriate intraoperative soft-tissue balance is essential for achieving successful outcomes following total knee arthroplasty (TKA). However, the optimal distraction force for evaluating joint laxity remains unclear. This study aimed to compare joint laxities measured using specific distraction forces with those obtained under manual varus-valgus stress and assess the interobserver variability.

**Methods:**

A total of 55 bi-cruciate-stabilized TKAs were performed using a robotic system. Medial and lateral joint laxities were evaluated using a force-controlled tensioner device under a distraction force of 60 and 80 N at 10°, 30°, 60°, and 90° of flexion and under manual varus-valgus stress using the robotic system. To assess interobserver differences, all measurements were performed independently by the senior surgeon and one of the assistant surgeons.

**Results:**

In the medial compartment, no statistically significant differences were detected between joint laxity under manual stress and that measured using a distraction force of 80 N at 10° and 30° of flexion. At 90° of flexion, no statistically significant difference was detected between JLR and JLT60N. In the lateral compartment, no statistically significant differences were detected between joint laxity under manual stress and that measured using 80 N at 10° and 90° of flexion, whereas joint laxity under manual stress was significantly greater than that measured using 80 N at 30° and 60° of flexion. Interobserver variability was greater in the lateral compartment than in the medial compartment, especially at higher flexion angles.

**Conclusions:**

The differences between joint laxity measured under manual stress and that measured using a tensioner device varied depending on the compartment and knee flexion angle. The lateral compartment showed greater measurement variability, suggesting the need for careful assessment and standardization of intraoperative joint laxity evaluation during robot-assisted TKA.

## Introduction

Achieving good clinical outcomes following total knee arthroplasty (TKA) requires appropriate soft-tissue balancing, as well as accurate osteotomy and implantation [1–3]. Several previous studies have reported that coronal stability during knee extension, mid-flexion, and 90° flexion can affect postoperative clinical outcomes and range of motion (ROM) [4–7]. Therefore, surgeons need to ensure appropriate soft-tissue balance during surgery. Measuring the joint gap using tensioner devices has been a widely used technique for evaluating intraoperative soft-tissue balance [8]. Another technique for evaluating intraoperative soft-tissue balance involves the use of computer-based tools, such as robotic systems and navigation techniques [9, 10].

Numerous studies have investigated the relationship between intraoperative joint laxity, measured by applying a distraction force using tensioner devices, and postoperative clinical outcomes. Accordingly, these studies have proposed several intraoperative joint laxity values that could be targeted to achieve favorable clinical outcomes [11, 12]. Although soft-tissue balance can be quantitatively evaluated using the tensioner device, similar quantification is difficult to achieve given that computerized assessments of soft-tissue balance require manual stress application [13]. Assessment of soft-tissue balance using a robot that requires manual stress application can produce highly variable results based on the force applied by the surgeon and the degree of knee flexion [14–17]. Therefore, clarifying the association between joint laxity measured using a specific distraction force and that measured under varus-valgus stress, as well as the reproducibility of measurements obtained under manual stress application, may greatly improve our understanding of the optimal soft-tissue balance required for successful treatment outcomes following robot-assisted TKA.

The current study aimed to compare joint laxities assessed under varus-valgus stress with those measured using distraction forces of 60 and 80 N across a range of flexion angles, as well as evaluate the presence of interobserver variability.

## Methods

This study was approved by the institutional review board. All patients provided written informed consent prior to participation.

From May 2024 to August 2025, 61 primary bi-cruciate-stabilized (BCS) (Journey II BCS, Smith and Nephew, Memphis, TN, United States) TKAs were performed by a single senior surgeon using a robotic system (CORI, Smith and Nephew, Memphis, TN, United States). Among them, 55 BCS TKAs were performed in 51 patients (10 males and 41 females; average age, 73.4 ± 7.8 years, range 54 to 85 years) who were included for the analysis. Six of the 61 TKAs were excluded because the component gap measurement device malfunctioned. Four patients underwent simultaneous bilateral TKA. Moreover, 53 and 2 knees were diagnosed with osteoarthritis and osteonecrosis, respectively. The average preoperative hip–knee–ankle (HKA) angle was 172.0 ± 7.7° (range 151°–190°).

### Surgical procedures

All patients underwent a paramedian approach with no eversion of the patella. The surgeon performed aggressive removal of osteophytes and minimal release of medial soft tissues for bone resection. In all cases, both the anterior cruciate ligament and posterior cruciate ligament were resected during surgical exposure. The patella was resurfaced with a polyethylene patellar component in all cases.

Femoral alignment was performed at a placement of 90° to the mechanical axis in the frontal plane and 4° of flexion in the sagittal plane using the robotics system. Tibial alignment was performed at a placement of 90° to the mechanical axis in the frontal plane and 4° of posterior slope in the sagittal plane.

The extension and flexion gaps were measured using a force-controlled compartment-specific ligament tensioner with a distraction force of 80 N each for the medial and lateral compartments (i.e., a total of 160 N) [18]. For posterior femur resection, the amount of tissue resected was adjusted to ensure that the extension and flexion gaps of the medial compartment remained equal to secure medial stability. Femoral rotation was parallel to the surgical epicondylar axis, allowing for residual lateral ligamentous laxity [19, 20]. Tibial rotational alignment was determined using the range of motion anatomical technique, thereby reducing the rotational mismatch of the components [21].

### Medial and lateral joint laxities evaluated using the tensioner device

**Fig. 1:**
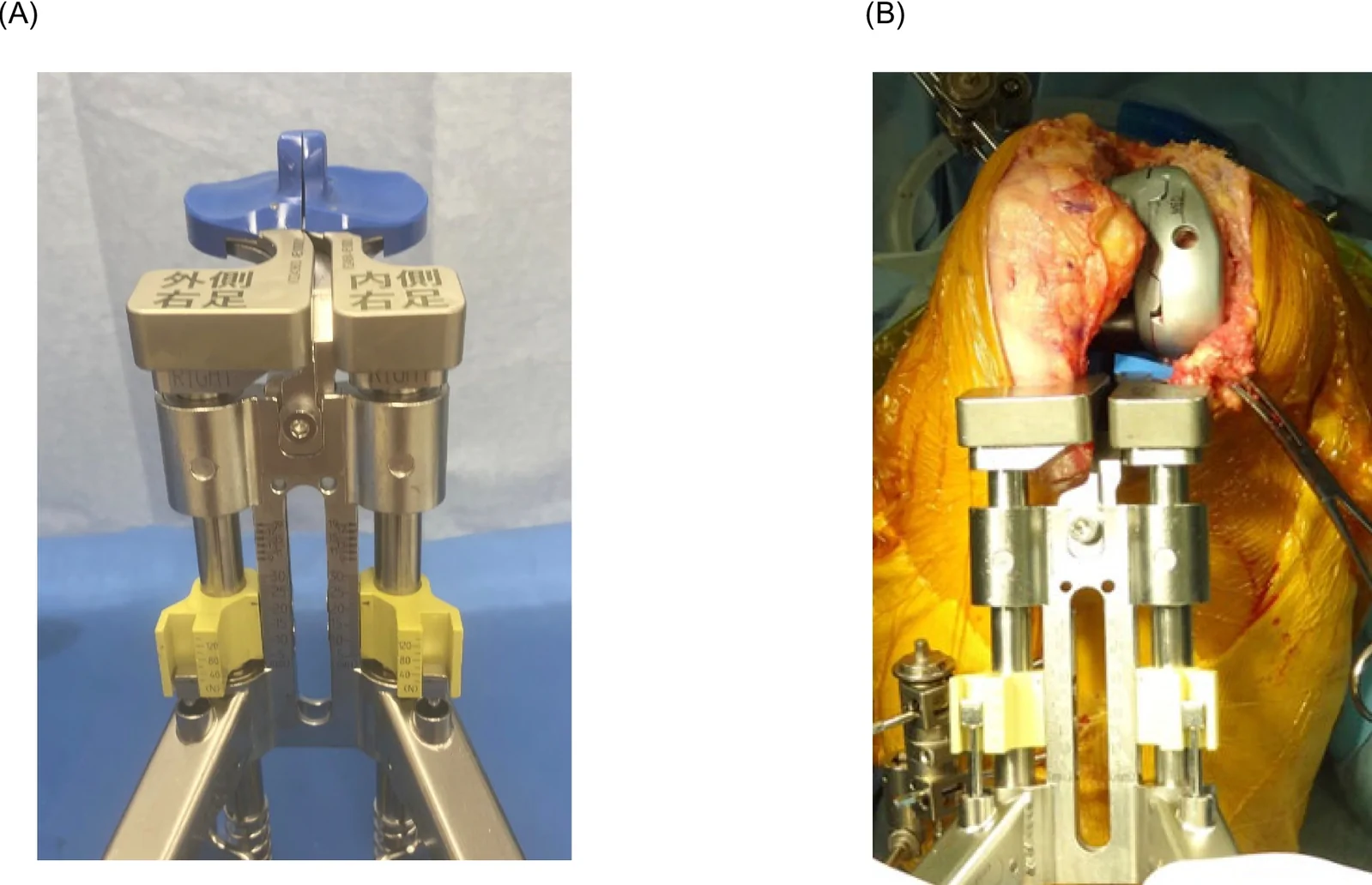
Force-controlled compartment-specific ligament tensioner. (**A**) The upper plates had identical shapes of medial and lateral compartments of the polyethylene trial surface of the Journey II BCS system. (**B**) Component gap measurement with the femoral trial implant in place

Component gaps were measured by the chief surgeon using a femoral trial implant and a force-controlled, compartment-specific ligament tensioner [22, 23], with distraction forces of 60 and 80 N for the medial and lateral compartments, at a knee flexion of 10°, 30°, 60°, and 90° (Fig. 1). The flexion angle was measured using the robotic system. The patellofemoral joint was reduced while the joint gap was decreased. Medial and lateral joint laxities evaluated using the tensioner device (JLT) were defined as the difference between the component gap value and the selected thickness of the tibial component.

### Medial and lateral joint laxities evaluated using the robotic system

**Fig. 2:**
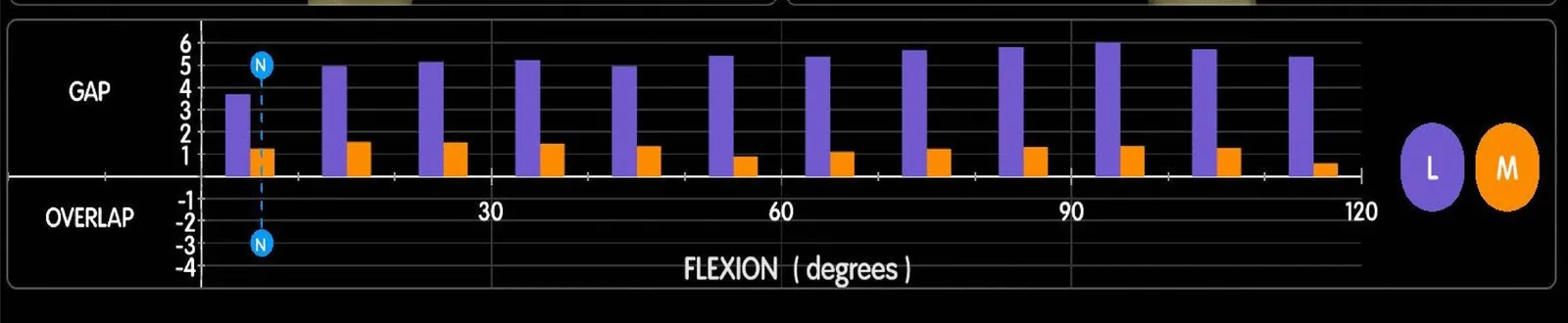
Measurement of joint laxity using the robot. The laxity results for both compartments are shown on the robot’s monitor

After prosthesis implantation, the medial and lateral laxities were evaluated by applying the maximum manual varus-valgus stress using the robotic system. First, the surgeon performed gentle, manual, and mild passive knee flexion without angular acceleration, while moving the leg from full extension to deep flexion. Next, using the maximum manual varus-valgus stress, the surgeon steadily moved the knee from full extension to flexion. Stress application and measurement cycles were repeated until the display registered no further changes in the recorded mediolateral joint laxity. The joint laxities of each compartment evaluated using the robotic system (JLR) were automatically shown on the display during the surgery (Fig. 2).

The measurement sequence was standardized for all cases. With the trial femoral and tibial components in place, joint laxity was first measured using a distraction force of 60 N in each compartment, followed by measurement using 80 N in each compartment. After implantation of the femoral and tibial components of the same size as the trial components, joint laxity was evaluated using maximum manual varus-valgus stress with the robotic system.

To evaluate the inter-measurer variability, measurements were performed by two surgeons, first by the senior surgeon (HI) and then by the first assistant surgeons consisting of three knee surgeons with moderate experience. They averted their gazes from the robotic system’s monitor during the senior surgeon’s evaluation to keep the measurements obtained by the senior surgeon unknown to them. The first assistants assessed 55 knees as follows: Assistant B assessed 28 knees, Assistant C assessed 21, and Assistant D assessed 6.

Interobserver agreement was assessed using a two-way random-effects, single-measure intraclass correlation coefficient for absolute agreement (ICC [2, 1]). The 95% confidence intervals were calculated using bootstrap resampling. Bland–Altman analysis was performed to calculate the mean difference (bias) and 95% limits of agreement (LoA), defined as the mean difference ± 1.96 standard deviations.

### Statistical analysis

Data were analyzed using the Bell Curve 2016 (SSRI Co., Ltd., Tokyo, Japan) software package for Microsoft Windows. Tests for normality and distribution were performed using the Kolmogorov–Smirnov test. Student’s paired t-test was used to compare the quantitative variables and differences between the two groups. A one-way repeated-measures analysis of variance (ANOVA) was performed to compare the JLR, JLT60N, and JLT80N. Post hoc pairwise comparisons were conducted using the Bonferroni correction. Power analysis was performed using G*Power 3 (Heinrich Heine University Düsseldorf, FRG). Based on a previous report [16], an α error of 0.05, a statistical power (1 − β error) of 0.80, and an effect size of 0.20 were used for the sample-size calculation. The resulting required total sample size was 42. All significance tests were two-tailed, with a *p*-value of < 0.05 indicating statistical significance for all tests.

## Results

**Fig. 3:**
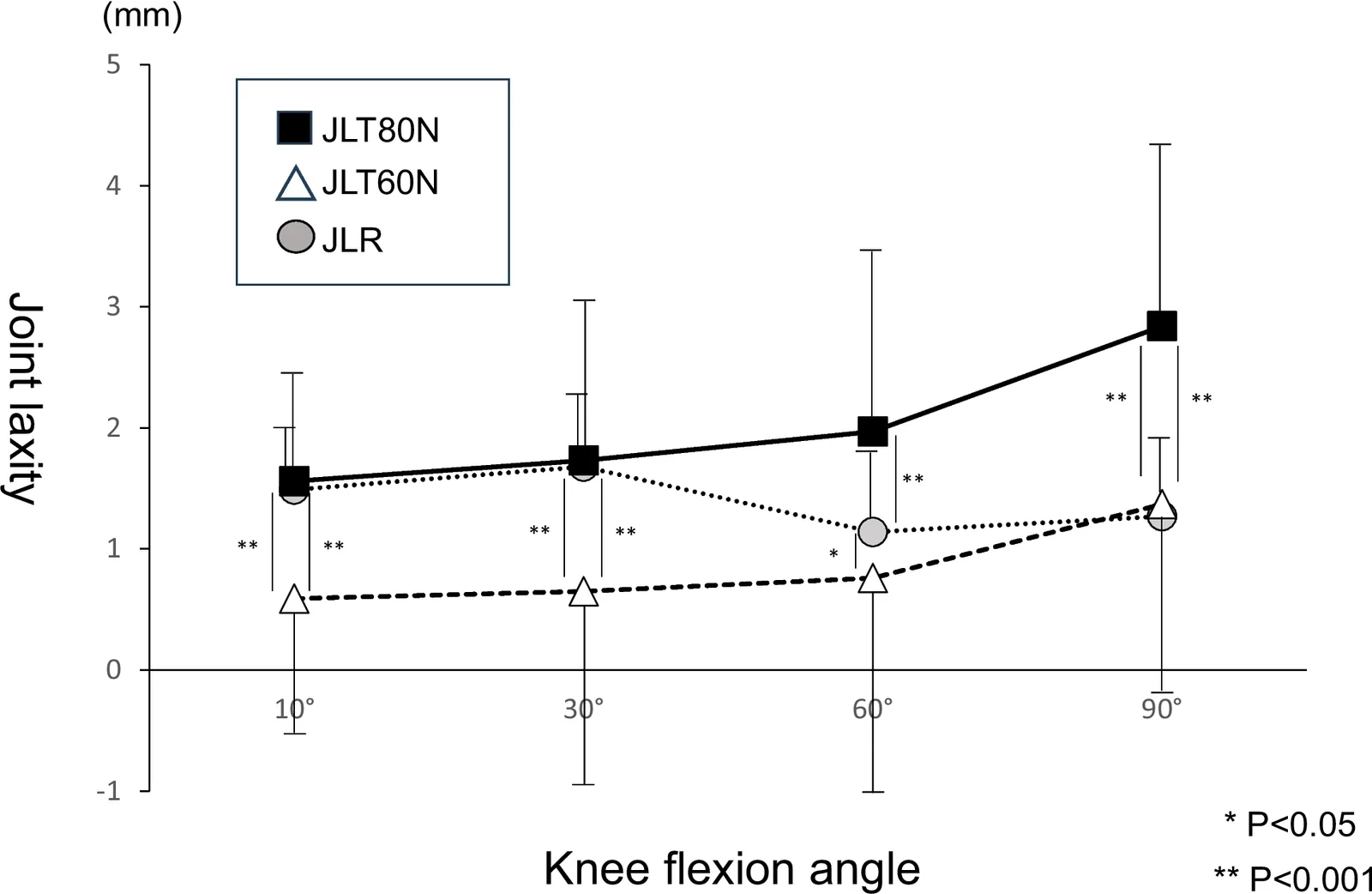
The comparison of the JLT60N, JLT80N, and JLR of the medial compartment. Error bars represent standard deviations

Figure 3 shows the JLT60N, JLT80N, and JLR of the medial compartment at 10°, 30°, 60°, and 90° knee flexion. At 10° flexion, JLR (*p* < 0.001) and JLT80N (*p* < 0.001) were significantly larger than JLT60N. At 30° knee flexion, JLR (*p* < 0.001) and JLT80N (*p* < 0.001) were significantly larger than JLT60N. No significant differences were observed between JLR and JLT80N at 10° and 30° knee flexion. At 60° knee flexion, JLT80N was significantly larger than JLR (*p* < 0.001) and JLT60N (*p* < 0.001), whereas JLR was significantly larger than JLT60N (*p* = 0.047). At 90° knee flexion, JLT80N was significantly larger than JLR (*p* < 0.001) and JLT60N (*p* < 0.001), whereas no significant difference was noted between JLR and JLT60N.

**Fig. 4:**
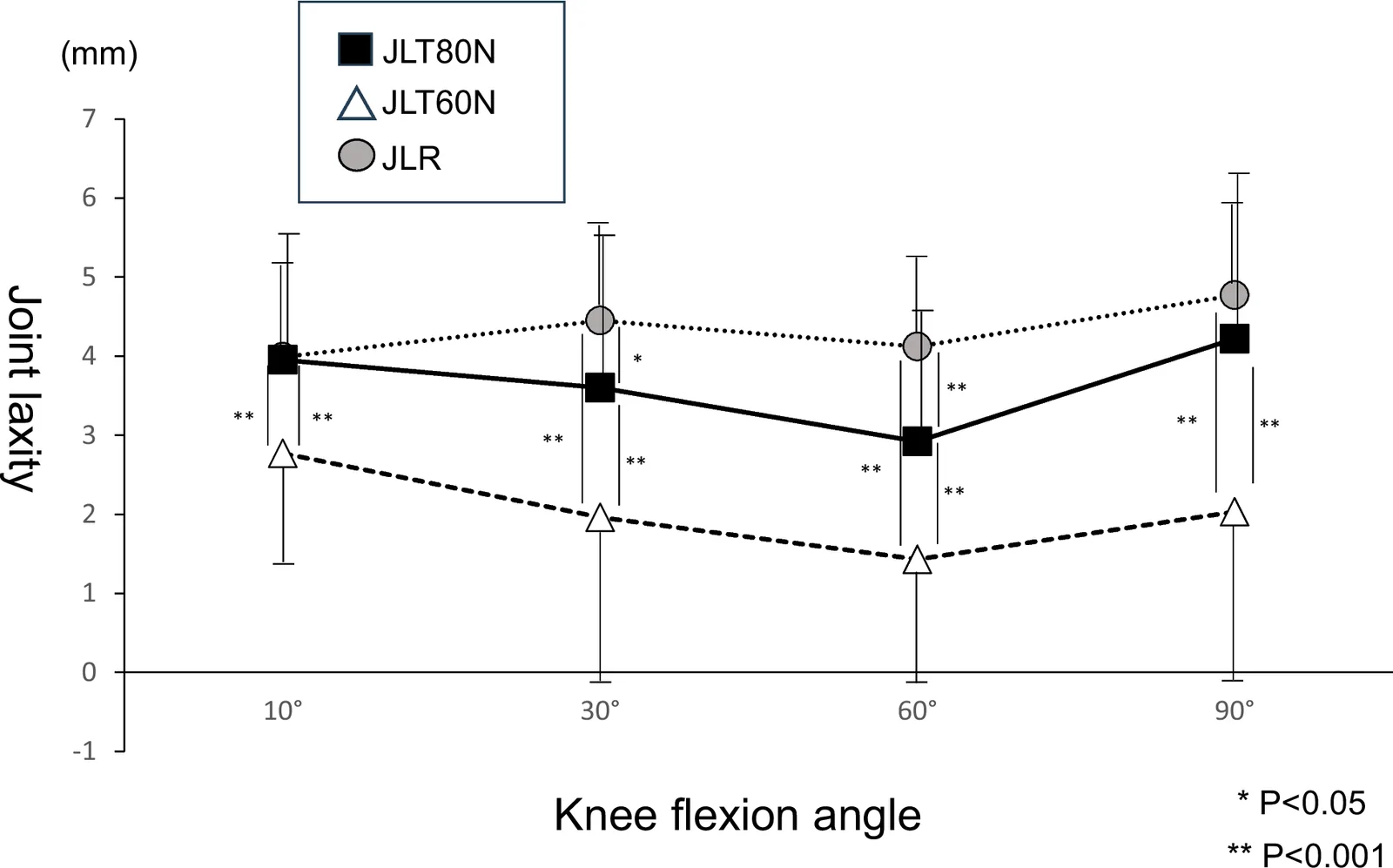
The comparison of the JLT60N, JLT80N, and JLR of the lateral compartment. Error bars represent standard deviations

Figure 4 shows the JLT60N, JLT80N, and JLR of the lateral compartment at 10°, 30°, 60°, and 90° knee flexion. At 10° knee flexion, JLR (*p* < 0.001) and JLT80N (*p* < 0.001) were significantly larger than JLT60N, whereas no significant difference was noted between JLR and JLT80N. At 30° knee flexion, JLR was significantly larger than JLT60N (*p* < 0.001) and JLT80N (*p* = 0.014), whereas JLT80N was significantly larger than JLT60N (*p* < 0.001). At 60° knee flexion, JLR was significantly larger than JLT80N (*p* < 0.001) and JLT60N (*p* < 0.001), whereas JLT80N was significantly larger than JLT60N (*p* < 0.001). At 90° knee flexion, JLR (*p* < 0.001) and JLT80N (*p* < 0.001) were significantly larger than JLT60N, whereas no significant difference was noted between JLR and JLT80N.

**Fig. 5:**
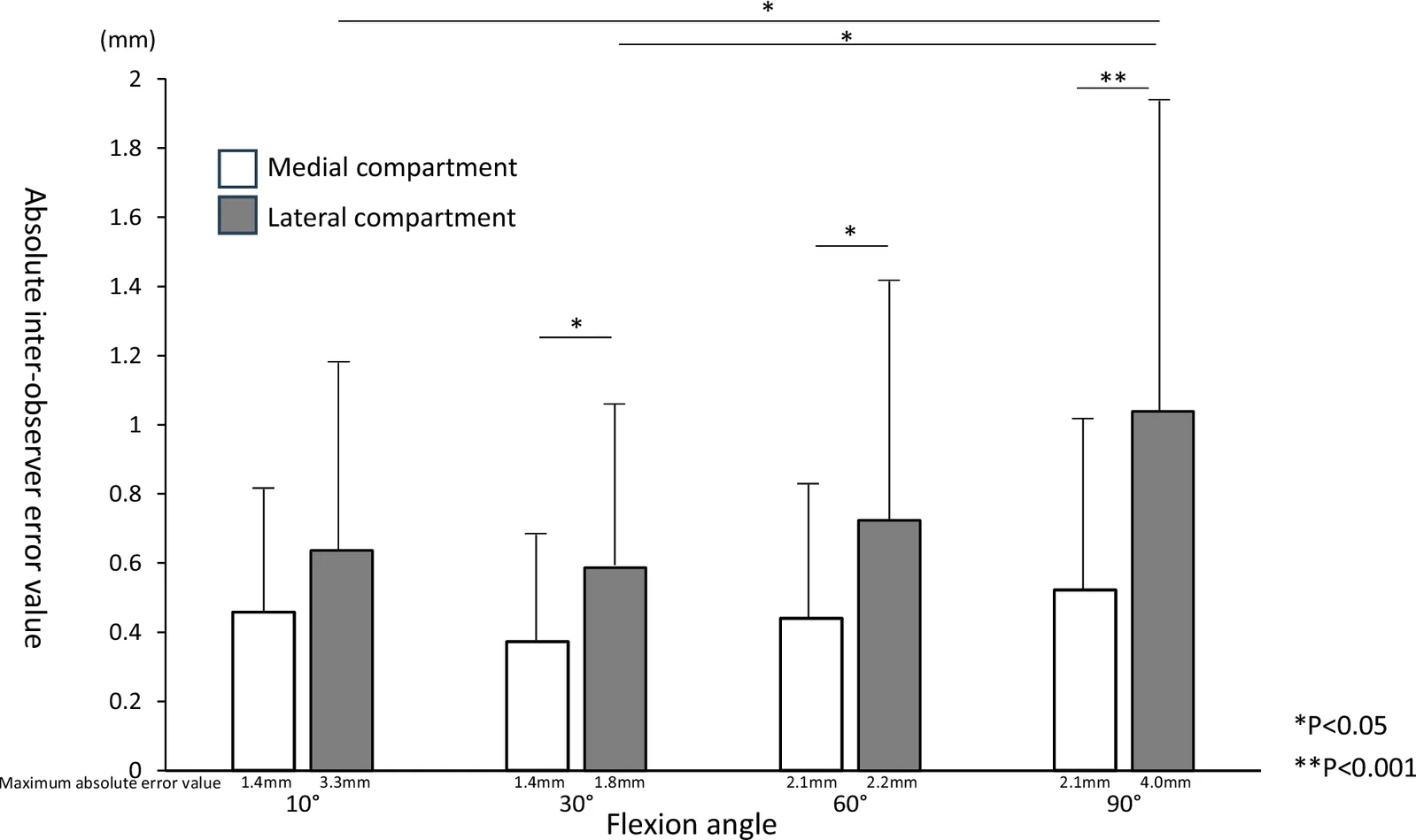
The absolute inter-observer error values at each flexion angle. Error bars represent standard deviations

Table 1 shows the JLRs evaluated by the senior surgeon and first assistants. Notably, no significant differences in any JLR parameters were observed between the two groups. Figure 5 presents the absolute inter-observer error values at each flexion angle. The absolute error values in the lateral compartment were significantly greater than those in the medial compartment at flexion angles of 30° (*p* = 0.010), 60° (*p* = 0.022), and 90° (*p* < 0.001). In the lateral compartment, the absolute error values at a flexion angle of 90° were significantly larger than those at 10° (*p* = 0.007) and 30° (*p* = 0.002), whereas no significant differences in absolute error values were observed among the flexion angles in the medial compartment.

**Table 1:** JLR evaluated by the senior surgeon and first assistants

	**Flexion angle**	**Senior surgeon**	**First assistants**	***p*-value**
Medial compartment	10°	1.5 ± 0.5 mm	1.5 ± 0.6 mm	0.792
	30°	1.7 ± 0.7 mm	1.8 ± 0.8 mm	0.284
	60°	1.1 ± 0.7 mm	1.2 ± 0.7 mm	0.267
	90°	1.3 ± 0.6 mm	1.3 ± 0.8 mm	0.601
Lateral compartment	10°	4.0 ± 1.3 mm	4.1 ± 1.4 mm	0.791
	30°	4.5 ± 1.3 mm	4.8 ± 1.4 mm	0.272
	60°	4.1 ± 1.3 mm	4.0 ± 1.7 mm	0.825
	90°	4.8 ± 1.3 mm	4.4 ± 1.8 mm	0.127

*JLR* joint laxity evaluated using the robotic system

Interobserver agreement was assessed between the senior surgeon and the first assistant using ICC analysis and Bland–Altman analysis. In the medial compartment, the ICC values at 10°, 30°, 60°, and 90° of flexion were 0.70 (95% CI 0.50–0.81), 0.77 (95% CI 0.66–0.86), 0.55 (95% CI 0.31–0.73), and 0.46 (95% CI 0.22–0.65), respectively. In the lateral compartment, the corresponding ICC values were 0.80 (95% CI 0.66–0.90), 0.84 (95% CI 0.76–0.90), 0.78 (95% CI 0.65–0.87), and 0.66 (95% CI 0.47–0.80), respectively. Bland–Altman analysis showed mean biases of − 0.03, − 0.14, − 0.14, and − 0.07 mm in the medial compartment and − 0.07, − 0.26, + 0.07, and + 0.48 mm in the lateral compartment at 10°, 30°, 60°, and 90° of flexion, respectively. The corresponding 95% limits of agreement were − 1.18 to 1.11, − 1.05 to 0.76, − 1.30 to 1.03, and − 1.52 to 1.38 mm in the medial compartment and − 1.78 to 1.64, − 1.72 to 1.15, − 1.96 to 2.09, and − 2.08 to 3.03 mm in the lateral compartment.

## Discussion

The most important findings obtained in the current study were that no statistically significant differences were detected between JLR and JLT80N at 10° and 30° in the medial compartment, whereas no statistically significant difference was detected between JLR and JLT60N at 90°. Additionally, inter-observer differences in the lateral compartment were larger than those in the medial compartment.

Knee joint stability in the coronal plane has been identified as a key factor for achieving successful treatment outcomes after TKA [24, 25]. Therefore, surgeons need to ensure appropriate soft-tissue balance during surgery through quantitative evaluation achieved using the tensioner device. However, the optimal distraction force to be used intraoperatively remains unclear. Asano et al. applied 60–180 N to the medial and lateral components during knee extension and flexion to investigate soft-tissue properties during posterior stabilized-type implants [26]. Accordingly, they reported stiffness values of 8.9 and 8.5 N/mm with a distraction force of 60 N during knee extension and stiffness values of 26.6 and 21.4 N/mm with a distraction force of 180 N during flexion. Moreover, they revealed that stiffness increased with distraction force. In contrast, Heesterbeek et al. showed that when forces higher than 60 N in each compartment (total of 120 N) are used for gap distraction, the ligamentous sleeve of the knee might experience over-tension [27]. Thus, the appropriate distraction force needed for gap measurements has remained controversial. One recent report showed that joint laxities evaluated under manual varus-valgus stress during robot-assisted surgery at extension showed no statistically significant difference from those measured under an 80 N/80 N distraction force using a tensioner device, whereas joint laxity values at other flexion angles were not equivalent [28]. The current study showed that no statistically significant differences were detected between joint laxities in the medial compartment measured under manual stress and those measured under 80 N using a tensioner device at 10° and 30° of flexion. At 60° of flexion, joint laxity measured under manual stress was significantly greater than that measured under 60 N, whereas no statistically significant difference was detected between the two measurements at 90° of flexion. These results could be attributed to the different behaviors of the capsular structures and collateral ligaments of the medial compartment at various knee flexion angles [29]. Surgeons should be aware that joint laxity evaluated using manual stress might be weak enough to underestimate the joint laxity evaluated using a tensioner device during flexion. These findings provide new information that may help determine appropriate distraction forces when assessing and adjusting soft-tissue balance during robot-assisted TKA.

Regarding the reproducibility of study results between surgeons, we found no significant interobserver difference in either the medial or lateral compartments when evaluated using the mean values. Somiya et al. reported that surgeon experience was a potent factor in the prediction of final soft-tissue balance [16]. When performing robot-assisted surgery, an experienced surgeon produces larger gaps using manual stress in both compartments when compared to an inexperienced surgeon. One of the reasons might be the extensive experience of the three first assistants in performing TKA, including robot-assisted TKA procedures. Also, Somiya et al. applied distraction forces to the medial and lateral compartments using manual stress via the Z-shaped retractor [16].

However, when evaluated using the absolute error values, the errors in the lateral compartment were greater than those in the medial compartment at flexion angles of 30°, 60°, and 90° and increased with greater degrees of flexion. A maximum inter-observer error of 4.0 mm was observed at 90° flexion in the lateral compartment. Although the lateral compartment possesses physiological laxity [20, 30], suggesting that a strict soft-tissue balance assessment may not be as necessary as that in the medial compartment, surgical concepts, such as kinematic alignment and functional alignment, which help optimize medial–lateral balance, are becoming more widespread [31, 32]. Similar to the findings of the current study, Corbett et al. reported that the reproducibility of lateral gap measurements in flexion was not excellent [33]. They attributed this limited reproducibility to the inherently greater compliance of the lateral ligamentous structures compared with the stiffer medial collateral ligament, which becomes more pronounced with increasing flexion [30], as well as to the technical difficulty of applying varus stress in the flexed position since application of varus force to the knee in flexion tends to externally rotate the hip [33]. The limited reproducibility of measurement of the lateral joint gap in flexion may be particularly relevant when performing functional alignment TKA using a robotic system. Inaccurate assessment of the lateral joint gap in flexion may potentially contribute to malrotation of the femoral component, postoperative restricted range of motion due to lateral joint tightness in flexion, or instability due to excessive lateral joint laxity in flexion. However, these potential clinical consequences were not directly evaluated in the present study and should therefore be regarded as hypotheses requiring further investigation. Therefore, surgeons performing functional alignment TKA using a robotic system should be aware that lateral gap measurements in flexion have limited reproducibility. Studies have reported that during BCS TKA, joint laxity in the lateral compartment at 90° flexion was associated with patient satisfaction [34]. Therefore, ensuring the reliability of lateral compartment evaluation has become increasingly important, warranting future investigations to improve its reproducibility.

### Limitation

Some limitations of the current study need to be discussed. First, varus–valgus stress was provided manually and was not quantified. Second, few patients were evaluated. Third, we failed to clarify the association between joint laxities evaluated using different methods and clinical outcomes. Fourth, the current analysis was performed using a single type of prosthesis (BCS type). Thus, the current results cannot be applied to other prostheses (i.e., posterior-stabilized, cruciate-retaining, or medial pivot type). Fifth, the current study did not evaluate the relationship between measurement variability of joint gaps and clinical outcomes. Sixth, the measurement order was fixed, with the senior surgeon always performing the first measurement followed by an assistant. Therefore, a potential order effect cannot be completely excluded. Seventh, the effects of repeated loading and tissue creep on joint laxity measurements could not be specifically evaluated in the present study because the measurement protocol was not designed to assess these effects. Therefore, the potential influence of repeated loading or tissue creep on the differences between JLT and JLR measurements cannot be excluded. Finally, four patients underwent bilateral TKA, and both knees from these patients were included in the primary analysis. A sensitivity analysis using one knee per patient, with the right knee selected for each of these four patients, showed no changes in the statistical significance of the results. However, the potential influence of within-patient correlation cannot be completely excluded.

## Conclusions

In the medial compartment, no statistically significant differences were detected between joint laxity evaluated under manual varus-valgus stress and that measured using a distraction force of 80 N at 10° and 30° of flexion, whereas no statistically significant difference was detected between JLR and JLT60N at 90° of flexion. In the lateral compartment, no statistically significant differences were detected between joint laxity evaluated under manual varus-valgus stress and that measured using a distraction force of 80 N at 10° and 90° of flexion, whereas the manual stress measurement was significantly greater than that measured using 80 N at 30° and 60° of flexion. Interobserver variability was greater in the lateral compartment, especially at higher flexion angles. These findings indicate that the differences between joint laxity measured under manual stress and that measured using a tensioner device vary according to the compartment and knee flexion angle, highlighting the need for standardized evaluation methods during robot-assisted TKA.

## Data Availability

The datasets used during the current study are available from the corresponding author on reasonable request.
